# Patterns of Healthcare Costs Among Older Adults: Demographics, Health Personality, and Resilience

**DOI:** 10.1093/geroni/igab046.3045

**Published:** 2021-12-17

**Authors:** Rotem Arieli, Peter Martin

**Affiliations:** Iowa State University, Ames, Iowa, United States

## Abstract

The purpose of this study was to examine healthcare costs of older adults in relation to demographic characteristics, individual health personality traits, and resilience. Data included 3,907 participants, 65 and older, collected by a large provider of Medicare Supplemental Health Insurance. The Health Personality Assessment*, Brief Resilience Scale, total healthcare cost, and demographic information were used. In our sample, the average healthcare cost was $13,283.69 (SD=30,784.87), ranging from $0–$989,084, and higher healthcare costs were found among older, male, and less health-neurotic (i.e., lower health-related anxiety) adults. Configural frequency analyses were conducted to identify “types” and patterns of healthcare costs by age and gender. The following significant patterns emerged: Women in the oldest group with high healthcare costs and women in the young-old age group who had low healthcare costs occurred significantly more than expected by chance, p<.01. Next, we hypothesized configuration patterns for resilience, health personality, and healthcare costs. Results confirmed the following “types” or patterns occurring more often than expected by chance: less-resilient individuals with high health neuroticism and high healthcare costs, p<.001, and less-resilient, less-health-conscientious adults with high healthcare costs, p<.001. The results suggest higher healthcare costs for individuals who are less resilient, more neurotic about their health, and less disciplined in their health practices. Future intervention programs may benefit from promoting resilience, reducing health neuroticism, and increasing health conscientiousness. *The Health Personality Assessment (HPA) is © 2021 United HealthCare Services, Inc. All rights reserved.

